# Nonlinearity- and Dispersion-Controlled High-Energy All-Fiber Femtosecond Laser System with Peak Power Exceeding 0.5 GW

**DOI:** 10.3390/nano16010032

**Published:** 2025-12-25

**Authors:** Feng Li, Qianglong Li, Jixin Xing, Xue Cao, Wenlong Wen, Lei Wang, Yufeng Wei, Hualong Zhao, Yishan Wang, Yuxi Fu, Wei Zhao

**Affiliations:** State Key Laboratory of Ultrafast Optical Science and Technology, Xi’an Institute of Optics and Precision Mechanics, Chinese Academy of Sciences, Xi’an 710119, China

**Keywords:** monolithic fiber femtosecond laser, high peak power, large dispersion, high-order dispersion compensation

## Abstract

A monolithic all-fiber high-energy chirped pulse amplification (CPA) system with a managed large dispersion is demonstrated. Considering the nonlinearity in the amplification system, two temperature-tuning cascaded chirped fiber Bragg gratings (CFBGs) with a large dispersion of 200 ps/nm are employed as stretchers to stretch the pulse duration to more than 2 ns in the time domain. The main amplifier, with centimeter-level length, a large mode area, and high-gain silicate glass fiber, increases the energy to 293 μJ at 100 kHz. A reflective grating pair with a high density of 1740 lines/mm is used to compress the large-dispersion chirped pulse into a compact structure. Owing to the high-order dispersion pre-compensation by the CFBGs and the large-sized grating with high diffraction efficiency, we achieved a compressed pulse duration of 466 fs with a maximum pulse energy of 250 μJ, corresponding to a compression efficiency of more than 85% and a well-preserved beam quality of M^2^ < 1.3. To the best of our knowledge, this is the highest pulse energy ever reported in a monolithic fiber femtosecond amplifier.

## 1. Introduction

Femtosecond lasers are favorable for a variety of applications, such as in the drilling of microholes, micro/nanofabrication, the welding of glass [1,2,3], and scientific research [4,5]. Many amplification methods have been proposed to achieve this target, with the most commonly used being thin-disk amplifiers [6,7], Innoslab laser amplifiers [8,9], traditional bulk crystal amplifiers [10], and fiber amplifiers [11,12,13,14]. Of all the amplifiers, monolithic all-fiber femtosecond lasers have shown great potential with their advantages including ease of integration and maintenance, good beam quality, and ease of heat dissipation. However, the inevitable intense nonlinear effects, such as self-phase modulation (SPM) and stimulated Raman scattering (SRS), limit the energy enhancement of fiber lasers because of the high peak intensity confined in a relatively small fiber core.

**Figure 1:**
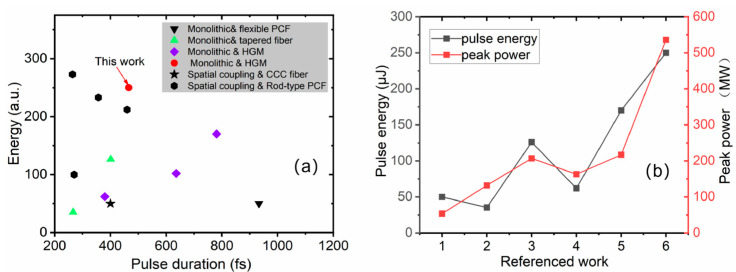
Reported high-energy fiber laser performances. (**a**) Pulse energy obtained from fiber femtosecond CPA laser systems; (**b**) energy and peak power from monolithic all-fiber femtosecond laser system.

In order to obtain high-pulse-energy ultrafast laser output in fibers, both sufficient temporal pulse stretching and the use of low-nonlinearity fibers in the spatial domain are vital. All kinds of large-mode-area fibers are employed in CPA systems to achieve a high energy output. A stretched pulse duration of ~600 ps and a flexible 40 μm core diameter photonic crystal fiber (PCF) achieved high energy output in a monolithic setup [15], with 50 μJ pulse energy and a compressed pulse duration of 933 fs. Researchers have presented a new type of fiber, a chirally coupled-core (CCC) fiber, to achieve single-mode output where the core size is larger than 30 μm. The pulse is stretched to 450 ps with 1 km polarization maintained on a single-mode fiber, achieving a maximum pulse energy of 50 μJ and a compressed pulse duration of 400 fs [16]. In recent years, tapered fibers have been used to achieve high-energy laser output. Even with 56 μm core diameter, the beam quality (M^2^) is still conserved in a single mode and can be better than 1.2 due to their multiclad structure and tapered design along the fiber length. In one chirped pulse amplification (CPA) system, a stretched pulse duration of ~1 ns was amplified in the tapered fiber with a repetition frequency of 2 MHz, ultimately achieving a pulse energy of 35 μJ with the narrowest pulse width of 266 fs [17]. Another chirped pulse amplification system used a similar tapered fiber with a wider stretched pulse duration of 1.7 ns, resulting in the laser output with a repetition rate of 504 kHz and a pulse energy of 126 μJ [18]. Among the high-gain medium (HGM) monolithic fiber amplifiers, by heavily doping and shortening the gain fiber length to ~20 cm, the accumulation of nonlinearity in CPA systems is greatly reduced. The seed pulse at a wavelength of 1.55 μm was first stretched to 1.4 ns before amplification and then amplified to 144 μJ at 100 kHz. Finally, after compression, approximately 70% of the pulse energy and the compressed pulse duration of 636 fs were observed in 2013 [19]. In the CPA system with a wavelength of 1.03 μm, the seed pulse was stretched to 1 ns before amplification and then amplified to 80 μJ at 400 kHz, as demonstrated in 2015 [20]. In our previous work, we achieved an energy value of more than 100 microjoules with the energy increased to 170 μJ and a pulse duration of 781 fs. However, the pulse duration of the main amplifier was restricted to approximately 1 ns after spectrum filtering [21]. In the high-energy fiber laser system, the rod-type PCF plays an important role due to its exceptionally large mode area. By using a one-stage flexible PCF and a one-stage rod PCF, a pulse duration of 270 fs with an average power of 100 W was obtained in 2016 [22]. By using a two-stage rod PCF, the compressed pulse duration of 357 fs and a pulse energy of 233 μJ were obtained in 2022 [23], and then a pulse energy of 212 μJ at a repetition rate of 0.5 MHz was obtained in 2024 [24]. Recently, a laser with an average power of 273 W and a pulse duration of 264 fs was achieved in a rod-type PCF [25]. Although the pulse energy of the femtosecond laser achieved with a rod-type PCF is satisfactory, spatial coupling, the non-bendable long rod structure (~1 m), and the high cost of the PCF itself pose significant challenges in large-scale applications. Alternative approaches have also been explored. A hybrid fiber–single-crystal fiber CPA system was also developed, emitting a pulse energy of 540 μJ and pulse width of 358 fs with 100 kHz [13], and an amplified pulse energy of 240 μJ at 1 MHz with the compressed pulse duration of 744 fs [26]. With coherent combination, the femtosecond fiber laser achieved a recorded pulse energy of 32 mJ at 20 kHz [27], and a 1 kW, 120 fs pulse [28] through 16 parallel ytterbium-doped rod-type amplifiers. Figure 1a summarizes the high-energy fiber femtosecond research results from [15,16,17,18,19,20,21,22,23,24,25] and this work, which demonstrate that the energy obtained in this work is the highest pulse energy obtained from monolithic femtosecond CPA all-fiber lasers and has the highest peak power; additionally, this energy level is equivalent to that of the rod-type PCF systems which require spatial coupling. Figure 1b gives the core parameters of energy and the peak power from a monolithic all-fiber femtosecond laser system.

We demonstrate a monolithic all-fiber femtosecond amplifier with a large stretch-compression ratio. The pulse is stretched to ~2 ns during the amplification stage with two large-dispersion CFBGs to reduce the nonlinearity accumulated in the amplification stage. The main amplifier is a heavily Yb-doped silicate glass fiber with a large mode area and a high gain within only a length of 20 cm. In this all-fiber amplifier, we obtained the maximum pulse energy of 293 μJ at 100 kHz. To compress the highly chirped pulse with enormous dispersion into a compact structure, a high-density 1740 lines/mm reflective grating pair is used. The optimization of high-order dispersion with temperature-tuning CFBGs results in a compressed pulse duration of 466 fs with a good beam quality. This high-energy monolithic all-fiber laser holds significant value in various applications.

## 2. Experimental Setup

**Figure 2:**
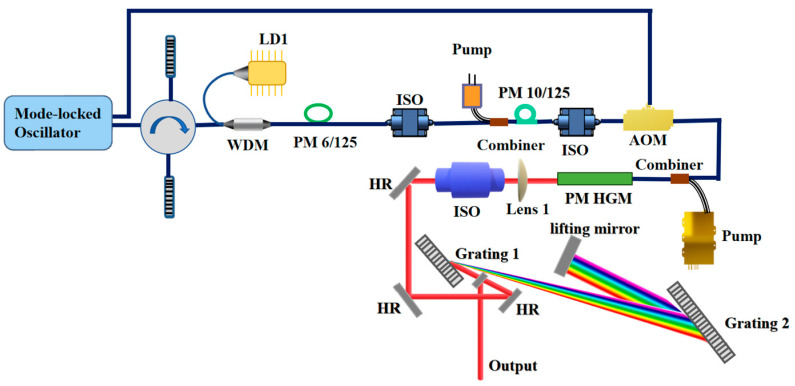
Experimental setup. LD: laser diode; WDM: wavelength division multiplexing; PM: polarization maintaining; ISO: isolator; AOM: acoustic-optic modulator; HR: high reflective; and HGM: high-gain medium.

Figure 2 shows the experimental setup for this all-fiber femtosecond laser. The CPA system includes a semiconductor saturable absorber mirror (SESAM) mode-locked seeder, a large-dispersion fiber stretcher, a single-mode (SM) fiber amplifier, a two-stage double-cladding fiber amplifier, and a pulse picker. Specifically, the main amplifier is a high-gain medium amplifier employing silicate glass fiber. After amplification, a large-aperture reflective grating pair with a high density serves as the compressor.

**Figure 3:**
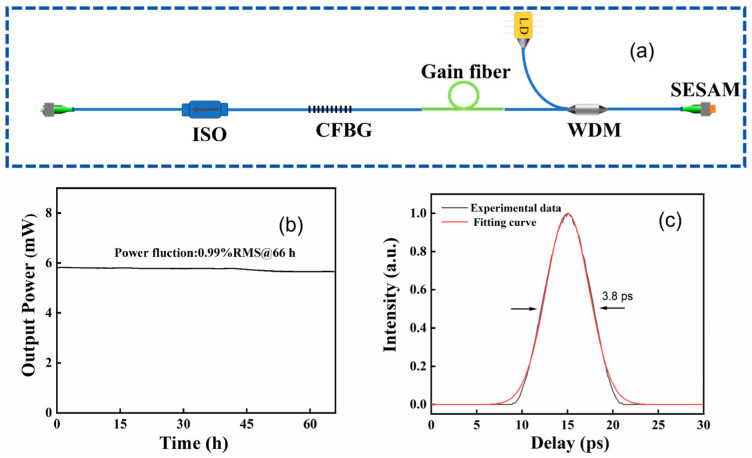
(**a**) The experimental setup of the seeder; (**b**) the power stability of the mode-locked seeder; and (**c**) the autocorrelation trace of the seeder.

The seeder is a home-built SESAM mode-locked fiber laser with a 34 MHz repetition rate and a power of 6 mW. The oscillator shows a spectral width of 12 nm with center wavelength of approximately 1030 nm. The schematic diagram of the mode-locked seeder is shown in Figure 3a. We conducted a continuous operation test over 66 h. During this period, the average output power demonstrated a remarkable power stability with a root mean square (RMS) value of 0.99%, which is shown in Figure 3b. This high degree of stability is crucial for the subsequent amplification stages. Regarding the temporal characteristics, the direct output from the oscillator exhibits a positive chirp, resulting in a measured pulse duration of approximately 3.8 ps, which is shown in Figure 3c. These pulses are readily compressible. Using a standard grating compressor, we have successfully compressed them to a duration of less than 300 fs, confirming their broad bandwidth and near-transform-limited quality. A circulator connects two temperature-tuning CFBGs, which are commercially available from Teraxion Co., Ltd., Quebec, Canada, to form the stretcher. The CFBGs have a reflection band of approximately 12 nm, thereby covering the full spectral bandwidth of the oscillator. The stretcher provides an ultralarge dispersion of 112.63 ps^2^ with a reflectivity of 49% over the operation bandwidth and can stretch the pulse duration to more than 2 ns to reduce the nonlinearity in the cascaded fiber amplifiers. The fabrication of CFBG, which serves as the core of the pulse stretcher, involves three main steps. (1) Hydrogen loading for photosensitivity enhancement: This pretreatment process significantly increases the fiber core’s susceptibility to ultraviolet (UV) light by diffusing molecular hydrogen into the photosensitive fiber. It is essential for achieving a strong and permanent refractive index change, especially in fibers with weak intrinsic photosensitivity. (2) UV laser phase mask inscription: The chirped grating pattern is written using a phase mask technique. A UV laser beam is shone through a chirped phase mask—a transparent plate with a surface relief grating whose period varies along its length. The interference pattern created by the diffracted beams induces a permanent, non-periodic modulation of the refractive index along the fiber core, replicating the mask’s pattern. (3) Post-writing annealing (hydrogen relief): Following inscription, the fiber undergoes a controlled annealing process. This step actively removes the residual hydrogen used in the first step, which permanently stabilizes the grating’s optical properties (such as reflectivity and central wavelength) and ensures its long-term reliability and performance consistency. The power after the stretcher is ~1 mW. An SM fiber amplifier is a 0.6 m gain fiber (Nufern, East Granby, CT, USA, PM-YSF-HI-HP) and is used to increase the power to approximately 10 mW. The following stage involves a 10/125 µm double-cladding (DC) amplifier (Nufern, PLMA-YDF-10/125-M) which is amplified to approximately 700 mW by a fiber-coupled 9 W multimode semiconductor pump laser. A polarization-maintaining (PM) DC fiber-coupled acousto-optic modulator (AOM) is subsequently employed to change the repetition rate from 34 MHz to 100 kHz. The average power after the AOM was about 1 mW. Subsequently, we employed another 10/125 DC amplifier to increase the power to 160 mW, which can satisfy the power needs of the final-stage main amplifier. The high-gain silicate glass fiber main amplifier is commercially available from Advalue Photonics, Tuson, AZ, USA, with fiber length of 20 cm and a large mode field diameter of 40 µm. It is conductively cooled on a water-cooled plate to maintain its high-power stability. The main amplifier employs a polarization-maintaining, high-gain medium (HGM), which is pumped by a 100 W, 976 nm fiber-coupled laser diode (LD) from BWT Beijing Ltd., Beijing, China, through a high-power (2 + 1) × 1 combiner. The pump scheme employed for this amplifier stage is a co-propagating configuration. This design helps optimize the overlap between the pump and signal modes, contributing to efficient energy extraction. With a pumped power of about 60 W, an amplified power of ~30 W is obtained. Due to the pump laser’s relatively wide beam divergence, a 5 mm aperture diaphragm was placed 15 mm from the fiber end face to filter out residual pump light. This simple pump power management scheme proves highly effective even at elevated pump powers of 154 W, ensuring an exceptional amplified output power stability of 0.105% at ~100 W [29]. Then, the amplified laser is collimated by a planoconvex lens with focal length of 50.8 mm and a half-wave plate is inserted into the optical path to obtain the optimal diffraction efficiency because of the polarization dependence of the reflective gratings. The compressor is a large-aperture reflective grating pair (commercially available from Plymouth Grating Laboratory, Carver, MN, USA) with a groove density of 1740 lines/mm, and high diffraction efficiency of more than 97%. The grating apertures are 110 mm × 110 mm and 210 mm × 110 mm, which is large enough to fully capture the diffracted spectrum without any loss of spectrum. The large aperture grating is also vitally important in our system. The manufacturing of large-aperture, high-quality gratings remains a significant technological hurdle, often described as a bottleneck technology for advancing high-power laser systems. The challenges are multifaceted and require balancing several competing demands, including high diffraction efficiency, broad bandwidth, scaling with high surface quality, high laser-induced damage threshold, and manufacture cost.

## 3. Results and Discussion

To generate high pulse energy from the monolithic all-fiber CPA system, the homemade linear cavity SESAM mode-locked laser has a wide spectrum of 12 nm (full width at half maximum, FWHM), which matches well with the stretcher reflection band of ~12 nm. To reduce the nonlinearity in the amplifier, the stretcher, which is composed of two large-dispersion CFBGs and a four-port circulator, provides a large dispersion of 112.63 ps^2^. Furthermore, the third-order dispersion pre-compensation is implemented using chirped fiber Bragg gratings (CFBGs). The primary source of third-order dispersion (TOD) is identified as the grating-pair compressor. The TOD introduced by the compressor was calculated using the following parameters: an input angle of 65.4°, a perpendicular grating separation of 970 mm, and a diffraction angle of 62°. Based on this configuration, the TOD of the compressor was calculated using the standard grating pair dispersion formula (1) to be approximately +1.5 ps^3^.

(1) $$\frac{d^{3}\varphi }{d\omega^{3}}=2\cdot \frac{3L_{g}\lambda^{4}}{4\pi^{2}c^{3}d^{2}}\cdot \frac{1+\frac{\lambda }{d}\sin\gamma -\sin^{2}\gamma }{\cos^{5}\theta }$$

where λ is the laser wavelength, L_g_ is the perpendicular separation distance of the grating pair, γ is the incident angle, d is the grating constant (groove spacing, related to groove density N by d=1/N), and c is the velocity of light in vacuum.

To achieve optimal pulse compression, it is essential to match the system’s second-order dispersion (group delay dispersion, GVD) while actively compensating for the TOD introduced by the compressor. Concurrently, the total dispersion accumulated in the fiber chain of the CPA system was evaluated. The system employs nearly 6 m of fiber, comprising both active (gain) and passive fibers. The fiber’s dispersion parameters are as follows: second-order dispersion (β_2_): 0.024764 ps^2^/m; third-order dispersion (β_3_): 46,000 fs^3^/m (or 0.046 ps^3^/m). Thus, the total dispersion accumulated over the 6 m fiber length is as follows: total GVD: ~0.149 ps^2^; total TOD: ~2.76 × 10^−4^ ps^3^ (negligible compared to the compressor’s contribution). Therefore, the third-order dispersion of −1.89 ps^3^ is pre-engraved into the stretcher of chirped fiber gratings. This value is slightly larger than the compressor’s +1.5 ps^3^, to account for potential system tolerances and ensure robust compensation. Additionally, the stretcher can offer SOD and TOD tuning capabilities of 4.5 ps^2^ and 0.5 ps^3^ by applying a temperature gradient along the CFBGs, which can achieve precise dispersion compensation matching with the compressor. The dispersion tuning principle with temperature is that, by applying a controlled temperature gradient along the CFBG, the local grating period and effective refractive index are altered via the thermo-optic and thermal expansion effects. This allows for post-fabrication and fine-tuning of the CFBG’s dispersion profile, enabling precise compensation of the residual third-order dispersion and ensuring high-quality pulse compression. The dispersion parameters, including the GVD and TOD, for the system are summarized in Table 1.

**Figure 4:**
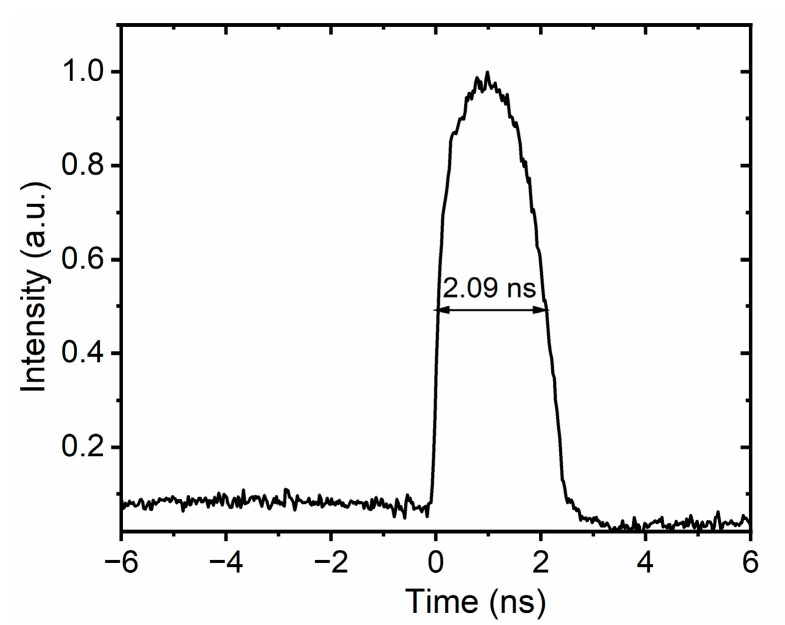
Pulse duration measurement after the stretcher.

After the stretcher, the mode-locked pulse is characterized using a high-bandwidth oscilloscope (Lecroy, 36 GHz bandwidth, 80 GS/s sampling rate), with the results shown in Figure 4. The pulse width (FWHM) is greater than 2 ns, and such a large pulse width is more conducive to increasing the pulse energy in the subsequent amplification process.

During the amplification process, the accumulated nonlinearities will directly affect the spectral and temporal performance [30,31]. Essentially, they can be quantified by the accumulated nonlinear phase shift in terms of the B integral and can be calculated as follows:

(2) $$B=\frac{\lambda }{2\pi A_{\mathrm{eff}}}\int_{0}^{L}n_{2}P_{\mathrm{AF}}(z)\mathrm{dz}+\frac{\lambda n_{2}}{2\pi A_{\mathrm{eff}}}P_{\mathrm{PF}}L_{\mathrm{PF}}$$

Here, λ is the laser wavelength, $n_{2}$ is the nonlinear refractive index coefficient, $P_{AF}(z)$ is the pulse peak power in the active gain fiber, L is the total fiber length, $P_{PF}$ is the pulse peak power in the passive fiber and can be assumed to be a constant value, $L_{PF}$ is the length of the passive fiber for transmitting the laser, and $A_{eff}$ is the fiber effective mode area. We assume that this process corresponds to a small signal amplification process in the active fiber and is constant in the passive fiber. In the fiber CPA system, the highest peak power is confined in the main amplifier. To compare the B integral in our main amplifier with the similar pulse energy obtained using a rod PCF with a large mode area reported in reference [23], the related calculation parameters and results are listed in Table 2. During amplification, the spectrum narrows due to the gain-narrowing effect. In the main amplifier of an HGM, the spectrum narrows to approximately 6 nm, with pulse duration of ~1.2 ns. According to the comparison, the B integral of the silicate glass fiber amplifier is only 1.38, which is significantly lower than that of the rod PCF of 4.38, making the silicate glass fiber amplifier more suitable for producing high-energy femtosecond laser outputs. Regarding the specific impact of SPM, the output spectrum of our amplifier shows only minor modulation and exhibits negligible broadening. This observation indicates that the SPM effect in our system is not severe. Spectral measurements reveal no evidence of stimulated Raman scattering (SRS) onset. The characteristic Stokes shift for our gain medium corresponds to an onset wavelength of approximately 1080 nm, yet this spectral feature is absent in the acquired data.

**Figure 5:**
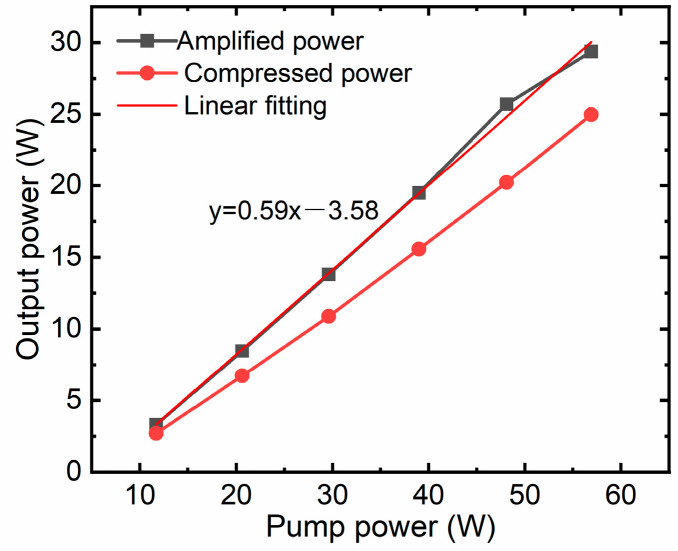
Output power performance vs. pump power.

Figure 5 shows the output power vs. the pump power. The power output of the amplifier is tested at various pump power levels, and through linear fitting, a high slope efficiency of 59% is attained. This high slope efficiency, along with the amplifier’s strong pump energy absorption capability, is primarily attributed to the use of a heavily doped gain fiber. As we know, to enhance the amplifier gain per unit length, it is necessary to increase the active ion concentration in the fiber through doping. However, higher doping concentrations lead to a reduction in the optimal fiber length. In silica-based large-mode-area (LMA) fibers, doping concentrations are constrained by strong ion clustering effects [32]. These effects arise from the close proximity of ions, which promotes cooperative energy coupling into parasitic transitions that compete with the desired laser transition. As a result, at high concentrations, ion–ion interactions reduce the upper laser level lifetime under increasing pump power, further diminishing amplifier efficiency. Unlike silica fibers, silicate or phosphate glass fibers can mitigate the detrimental effects of high rare-earth ion concentrations. By employing heavily doped silicate glass fibers, sufficient pump absorption is achieved while maintaining relatively high slope efficiency.

**Figure 6:**
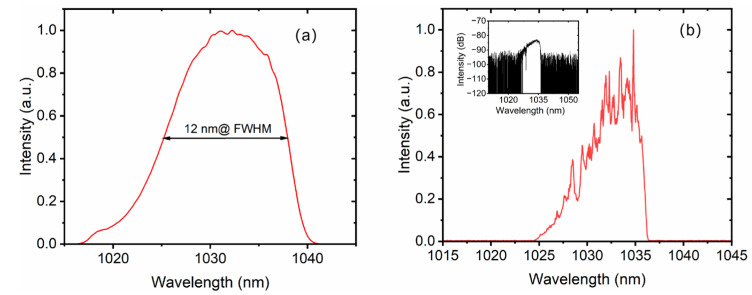
Spectrum of the laser. (**a**) Spectrum of the seeder oscillator; (**b**) spectrum of the compressed laser. Inset: the spectrum of amplified laser in logarithmic.

**Figure 7:**
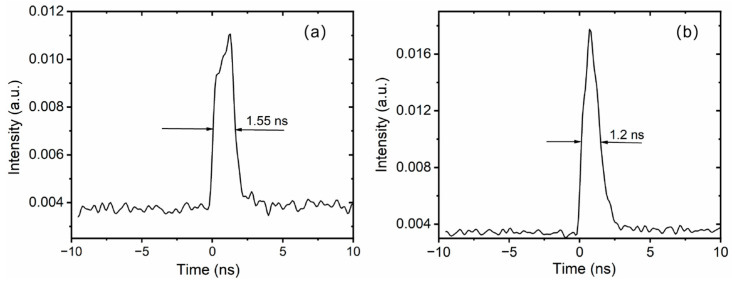
Oscilloscope trace of amplified pulses. (**a**) Pulse traces at 10 W; (**b**) pulse traces at maximum power.

Notably, the signal injected into the main amplifier is only 160 mW, whereas the maximum output power reaches 29.3 W at 100 kHz, corresponding to a high gain of 1.13 dB/cm. Scaling to significantly higher pulse energies within this specific configuration presents a formidable challenge. This limitation stems primarily from two factors: the risk of peak-power-induced fiber damage and the onset of nonlinearity-induced pulse distortion. Conversely, when operating the system at a higher repetition rate—specifically, 1 MHz—we demonstrated an average power output of approximately 100 W. This regime shifts the primary limiting factor from nonlinearities to thermal dissipation. By using a large-aperture reflective grating pair with a high groove density of 1740 lines/mm to compress a laser pulse with large dispersion, a maximum compressed pulse energy of 250 μJ is obtained, corresponding to a total compression efficiency of 85.3%. The optimal theoretical compressor efficiency, considering only the grating losses, would be (0.97)^4^ ≈ 88.5%. In practice, we must also account for the losses of the reflective mirror and the lifting mirror. Considering these additional losses, the measured total compressor efficiency of 85.3% is within the normal and expected range for such a high-performance system. The spectrum of the compressed pulse is also measured by the optical spectrum analyzer, Yokogawa AQ6370D (Tokyo, Japan), with a spectrum width of approximately 6 nm (FWHM), as shown in Figure 6b, which supports a short-pulse-duration output. The spectrum has some modulations, which are caused primarily by the nonlinear effect of SPM. There is no spectrum filtering, indicating that the aperture of the reflective grating is large enough to receive the entire spectrum. The seeder oscillator spectrum is also measured, as shown in Figure 6a, which has a spectrum width of 12 nm. The Yb-doped fiber gain medium exhibits a relatively weaker gain narrowing effect compared to solid-state laser media like Yb:YAG. This is due to the significantly broader emission bandwidth of Yb-doped fibers. Consequently, after amplification and compression, a final spectral width of 6 nm is obtained, as shown in Figure 6b. The logarithmic-scale spectrum is presented in the inset of Figure 6b, demonstrating negligible amplified spontaneous emission (ASE) components within the measurement sensitivity. Although high-energy Yb-doped solid-state lasers, such as the Yb:YAG laser, have lower nonlinearity and higher pulse energy output, they suffer from significant gain narrowing during high-gain amplification, resulting in a narrow spectrum width of typically less than 3 nm and pulse widths greater than 700 fs [33,34]. To assess the pulse evolution of the amplifier, the pulse widths are measured, as shown in Figure 7, and with increasing amplification power, the gain narrowing effect becomes progressively more pronounced. This phenomenon manifests as a continuous spectral narrowing, while the pulse duration simultaneously decreases due to the chirped nature of the pulses. As illustrated in Figure 7, the pulse duration decreases from approximately 1.55 ns at an amplified power of 10 W to about 1.2 ns at maximum output power of 29.3 W.

**Figure 8:**
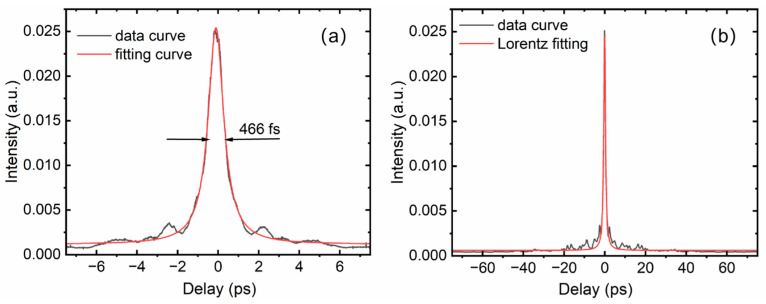
Pulse duration measurement. (**a**) Autocorrelation curve in the 15 ps scanning range; (**b**) autocorrelation curve in the 150 ps scanning range.

**Figure 9:**
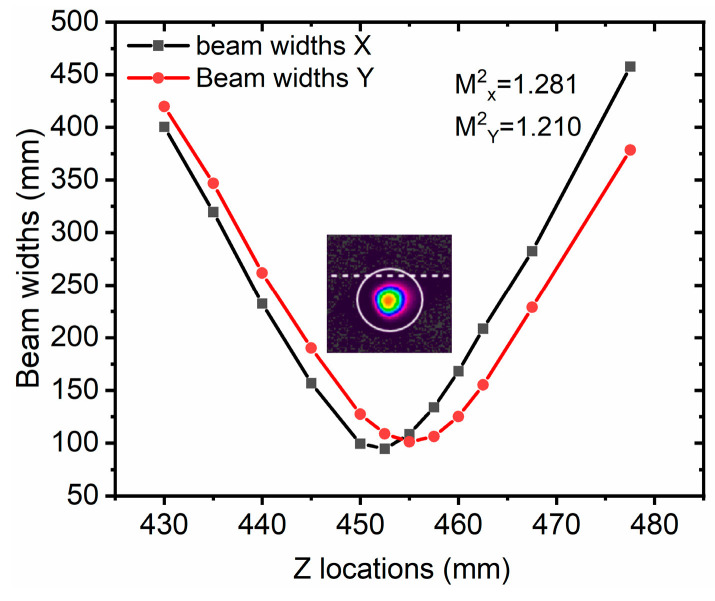
Beam quality measurement.

To obtain a short-pulse-duration output, the distance of the grating pair and the input angle of the grating are carefully adjusted. The shortest pulse duration was measured by the autocorrelator at a linear distance of approximately 2.1 m for the grating pair, which results in a second-order dispersion of 112.5 ps^2^. To achieve the optimal short pulse duration, we use temperature tuning of the CFBGs to achieve more precise dispersion matching. The third-order dispersion of −1.89 ps^3^ is pre-engraved into the stretcher of the CFBGs, which can compensate for the TOD of the grating pair. The optimal distance of 2.1 m results in a third-order dispersion of ~1.5 ps^3^ for the compressor. This value is estimated using an input angle of 65.4° and a diffraction angle of 61.9°, which differs from the Littrow angle for beam splitting. The third-order dispersion still has a slight mismatch with the stretcher, and the compressed pulse duration cannot reach the transform-limited pulse duration. The compressed pulse duration is measured by the APE pulsecheck autocorrelator. As shown in Figure 8, the pulse duration is 466 fs with Lorentz fitting in a 15 ps scanning range. When different pulse fittings of Gaussian, Sech^2^, and Lorentz fitting are used, we find that the Lorentz fitting method is the closest to the actual pulse shape. To evaluate the energy distribution, we also scan the pulse in a 150 ps range, and we can observe that the pulse has a small pedestal. For most applications, beam quality is crucial. The monolithic all-fiber CPA system, based on a silicate glass fiber, exhibits good beam quality. As shown in Figure 9, by carefully adjusting the grating pair, the beam quality is still conserved in single mode, with a beam quality better than 1.3, which is measured by the beam quality analyzer of Ophir beam squared SP920, Jerusalem, Israel. In the measurement of beam quality, the output beam was attenuated using a high-quality beam splitter. Only approximately 0.5% of the total beam power was sampled for the characterization. This approach serves two critical purposes: first, it ensures the safety of the sensitive detector within the beam profiler by preventing potential saturation or damage from high optical power; second, it meets the input power range requirement of the measurement equipment.

## 4. Conclusions

In this work, an all-fiber high-energy femtosecond fiber laser based on chirped pulse amplification technique is demonstrated. To mitigate nonlinear effects during amplification, the seeder pulses are stretched to ~2 ns for the subsequent fiber amplification. The main amplifier, a short-length silicate glass fiber with a large mode area and a high gain, increases the energy to 293 μJ at 100 kHz, with an amplification gain of 29.6 dB. To construct a compact compressor, a large-aperture reflective grating pair with a density of 1740 lines/mm is used as the compressor. Due to the temperature tuning of the stretcher, the SOD and TOD are simultaneously compensated and a short pulse duration of 466 fs is obtained, corresponding to a peak power of 536 MW. This monolithic all-fiber femtosecond laser will show wide value in various applications.

## Figures and Tables

**Table 1:** Details of the dispersion control in the monolithic all-fiber CPA system.

Components	Dispersion	Designed or Calculated
Stretcher	Second-order dispersion of CFBGs	112.63 ps^2^
	Third-order dispersion of CFBGs	−1.89 ps^3^
	Tuning range of second-order dispersion	≥4.5 ps^2^
	Tuning range of third-order dispersion	≥0.5 ps^3^
Fiber dispersion in CPA	Second-order dispersion of fiber	~0.149 ps^2^
	Third-order dispersion of fiber	2.76 × 10^−4^ ps^3^
Compressor	Linear distance of the grating pair	210 cm
	Second-order dispersion of the grating pair	−112.5 ps^2^
	Third-order dispersion of the grating pair	1.5 ps^3^

**Table 2:** B integral calculation comparison of a Rod PCF and a silicate glass fiber.

	Fiber Type	In and Out Energy (μJ)	Length (mm)	MFD (μm)	τ (ns)	B (rad)
Ref. [23]	Rod PCF	33 & 333	800	60	1.5	4.38
This work	Silicate glass fiber	1.6 and 293	200	40	1.2	1.38

MFD, mode field diameter; τ, pulse duration.

## Data Availability

The data that support the findings of this study are available from the corresponding authors upon reasonable request.
